# A New Approach Utilizing Aza-Michael Addition for Hydrolysis-Resistance Non-Ionic Waterborne Polyester

**DOI:** 10.3390/polym14132655

**Published:** 2022-06-29

**Authors:** Hao Fu, Linbo Gong, Shuling Gong

**Affiliations:** College of Chemistry and Molecular Sciences, Wuhan University, Wuhan 430072, China; haofu@whu.edu.cn

**Keywords:** aza-Michael addition, comb-like polymer, non-ionic waterborne polyester, hydrolysis resistance

## Abstract

This work first synthesized a series of linear polyesters by step-growth polycondensation, then an amino-terminated hydrophilic polyether was grafted to the polyester as side-chains through aza-Michael addition to prepare a self-dispersible, non-ionic waterborne comb-like polyester (NWCPE). In contrast to traditional functionalization methods that usually require harsh reaction conditions and complex catalysts, the aza-Michael addition proceeds efficiently at room temperature without a catalyst. In this facile and mild way, the NWCPE samples with number-average molecular weight (*M*_n_) of about 8000 g mol^−1^ were obtained. All dispersions showed excellent storage stability, reflected by no delamination observed after 6 months of storage. The NWCPE dispersion displayed better hydrolysis resistance than an ionic waterborne polyester, as was indicated by a more slight change in pH value and *M*_n_ after a period of storage. In addition, the film obtained after the NWCPE dispersion was cross-linked with the curing agent, it exhibited good water resistance, adhesion, and mechanical properties.

## 1. Introduction

The emission of volatile organic compounds (VOC) is harmful to the environment and human health, causing many countries and regions have formulated strict restrictions on it. This has driven the development of waterborne polymer materials [1]. In addition to the characteristics of traditional polyester (including high gloss, good weather resistance, and biocompatibility), waterborne polyester (WPE) also has the advantages of low VOC, low toxicity, lack of odor, nonflammability, ease of disposal, and so on [2,3]. These characteristics make waterborne polyester attract lots of attention in coatings, adhesives, biomedical applications, and other fields [4,5,6].

According to whether an emulsifier is used in the preparation process, WPEs can be divided into two groups: external-emulsifying polyester and self-emulsifying polyester. The emulsifiers are required in the preparation of external-emulsifying WPE. There are often residual emulsifiers in the prepared emulsion which would migrate to the surface of the emulsion in the film-forming process. This event would affect the performance of emulsions, as well as reduce the surface evenness and the water resistance of cross-linked films [7]. Self-emulsifying WPE can be roughly divided into ionic and nonionic types according to their hydrophilic components. The ionic WPE usually contains carboxyl groups or sulfonic acid groups. When Ma et al. [8] prepared WPE with trimellitic anhydride as terminal groups, the polyester dispersed well in water after neutralization, but the molecular weight was below 2000 g mol^−1^, which would limit its application scope. Zhang et al. [9] introduced 5-sulfoisophthalic acid monosodium salt into the molecular backbone and obtained a WPE with a solid content of 10%. Although the ionic polyesters are facile in synthesis and dispersing procedures, there are still defects to be resolved. The anionic polyester is usually difficult to apply in acidic environments and has poor compatibility with certain additives and inert pigments, [10] and the sulfonate-based polyesters are corrosive to metal substrates [11]. Waterborne polyester prepolymer with a molecular weight of around 6000 g mol^−1^ usually exhibits better comprehensive properties and is suitable for the classical hardening formulations, [12] but it was difficult for ionic WPE to achieve such a molecular weight. For the carboxyl-terminated polyester, decarboxylation is unavoidable at high temperatures; moreover, only terminal carboxyl groups cannot provide sufficient hydrophilic capacity for high molecular weight polyester [13]. For the multi-carboxyl containing polyester, the uncontrolled branching reaction between carboxyl groups and polyols would limit the growth of molecular weight [14]. For the sulfonate-based polyester, the molecular weight of polymer decreases when a sulfonic acid monomer is involved in copolymerization [15]. Moreover, ionic WPEs are usually poor in hydrolysis resistance. Non-ionic WPEs, which rely on polyethylene glycol (PEG) segments on the main chain to achieve water-dispersibility, would sacrifice the hydrolysis resistance and mechanical properties, as well as the hydrophilicity of the obtained polymer is usually weak. [16,17]. 

Therefore, researchers have made some attempts to explore other methods for the preparation of water solubility polymers [18]. It was found that by grafting the hydrophilic segments as side chains to the molecular backbone, the molecular weight of the WPE was improved, as was the mechanical property, etc. [19,20] Li et al. [20] used dimethylolpropionic acid modified by PEG to synthesize a polyester prepolymer. After conducting a reaction with isocyanate, a series of non-ionic waterborne comb-like polyurethane were obtained. With the increase in the comb-like polyester segment, the elongation at break (ε) of the product increased by 30%, and the solid content increased from 20% to 30%. However, the modification process has to undergo tedious protection and deprotection procedures. Taniguchi et al. [21] prepared a water-soluble, comb-like polyester through a chemoselective reaction between PEO side chains and ketone-bearing polyester; however, the water-solubility and mechanical properties of these polymers were not discussed in detail, and the synthetic pathways were complex, involving multiple steps with multiple catalysts. Furthermore, when side chains with a certain steric hindrance were grafted to the molecular skeleton to form a comb-like structure, the ester bonds of the polyester were protected according to the “rule of six” theory, and the hydrolysis resistance of the WPE would be improved [22]. However, the synthesis of polyesters with side chains or side chain functionalized groups usually require complex multistep reactions under harsh conditions including multiple expensive catalysts and long reaction times, which usually leads to the degradation of polyester chains in deprotection reactions [21,23]. In recent years, the aza-Michael addition, which is considered as a very efficient method to create new C-N bond and β-amino carbonyl derivatives, has attracted the attention of researchers in the post-polymerization modification of polymers [24,25,26]. The aza-Michael addition proceeds between a nucleophilic amine and an electron-deficient alkene. Interestingly, dimethyl maleate, a common structural unit in polyester, has been confirmed to be a class of Michael receptors with high reactivity and selectivity [27]. Bosica et al. [28] demonstrated that dimethyl maleate could undergo an efficient Michael addition reaction with various aliphatic amines at room temperature without any catalyst. Yu et al. [29] also confirmed that the primary amines with different chain lengths react efficiently with *cis*-methyl maleate and that the obtained sample was highly functionalized, with the addition reaction being almost irreversible. These works inspire us for preparing NWCPE via green aza-Michael addition.

This work aims to develop a facile and green methodology for preparing self-dispersible NWCPE. By step-growth polycondensation and aza-Michael addition, the NWCPE with hydrophilic polyether-amines Jeffamine M-1000 as side chains was prepared. In this way, the properties of waterborne polyester, such as molecular weight, storage stability, hydrolysis resistance, and mechanical properties have been improved. The results exhibited that the NWCPE has excellent application prospects in eco-friendly waterborne coating.

## 2. Materials and Methods

### 2.1. Materials

Maleic acid (MA), hexanedioicacid (HA), n-butyl titanate (Ti(OBu)_4_), methanol, chloroform, and p-toluene sulfonic acid (TsOH) were obtained from Sinopharm Chemical Reagent Co., Ltd. (Shanghai, China). In addition, 1,4-cyclohexanedimethanol (CHDM), 1,6-hexanediol (HG), eopentyl glycol (NPG), and dibutyltin dilaurate (DBTDL) were purchased from Aladdin Reagents Co., Ltd. (Shanghai, China). Jeffamine^®^ M-1000 was commercialized by Huntsman Corp. (Texas, USA). Waterborne non-ionic hexamethylene diisocyanate trimer (WHDIT, with a -NCO content of 19.5 ± 0.5%) was purchased from Habo Chemical Co., Ltd. (Wuhan, China).

### 2.2. Methods

#### 2.2.1. Synthesis of Linear Polyesters

The mixture of monomers (MA, HA, HG, CHDM, and NPG), catalyst (0.6 mol% with respect to the diacids), and antioxidant 4-methoxy phenol (0.5 wt% related to all reactants) were added to a 100 mL three-neck round-bottom flask, which was equipped with a mechanical agitator, a nitrogen inlet, and a condenser. Firstly, the mixture was stirred at 180 °C (760 mmHg) under N_2_ atmosphere for 5h. Secondly, the pressure of the system was gradually decreased to 0.3–2 mmHg, and the mixture was stirred under vacuum until an apparent Weissenberg effect (the viscoelastic liquids climb up to a rotating rod) was observed. The product was dissolved in chloroform, followed by precipitation with a large amount of cold methanol. Following this, a pale yellow, transparent, viscous, linear polyester was obtained. 

#### 2.2.2. Synthesis of Comb-like Polyester

**Scheme 1:**
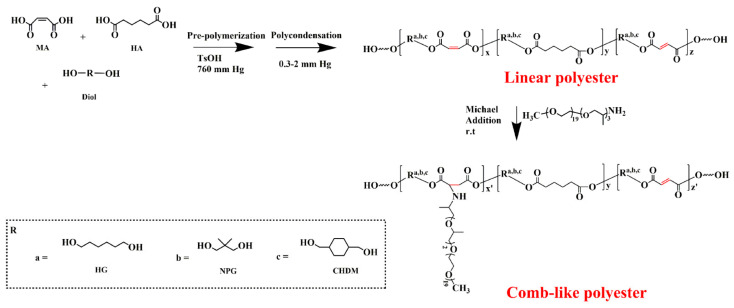
The synthesis route of comb-like polyester.

Once the linear polyester was dissolved in chloroform, the Jeffamine M-1000 was added to the solution at a ratio of 10:3 ([maleate units]/[Jeffamine], mol/mol), and the mixture reacted at room temperature for 10 h. The mixture was precipitated by being poured into diethyl ether and was then dried in a vacuum oven. The obtained comb-like polyester was a light yellow fluid and its viscosity was lower than the linear polymer prepared in previous step. The synthesis route of comb-like polyester was displayed in Scheme 1.

#### 2.2.3. Preparation of Non-Ionic Waterborne Comb-like Polyester (NWCPE) Dispersion

The dried comb-like polyester was dispersed by adding deionized water dropwise at 60 °C under a mechanical dispersion process at 1500 rpm, after which a light yellow and transparent NWCPE dispersion with 40% solid content was obtained. The formulations of different linear polyester and NWCPEs were listed at Table 1.

#### 2.2.4. Cross-Linking of the NWCPE Dispersions

The synthesized NWCPE dispersion and curing agent WHDIT were fed in a ratio of -NCO/-OH = 1.5:1.The mixture was diluted to solid content of 35% with deionized water under stirring. The mixture was stirred for 15 min and then coated on a glass plate or clean tinplate After drying to constant weight at room temperature, the film was taken out for further testing.

### 2.3. Characterizations

Fourier transform infrared spectroscopy. FTIR of samples was performed by Thermo Nicolet Fourier transform infrared spectroscopy (Thermo Fisher, Waltham, MA, USA) with the attenuated total reflectance (ATR) method. The scanning range was 4000–500 cm^−1^.

Nuclear magnetic resonance. ^1^H NMR spectra of samples were taken on a Bruker Avance-400 MHz spectrometer in deuterated chloroform.

Particle size. Malvern Zetasizer Nano Model ZS90 was used to measure the particle size and distribution (PDI). The sample was diluted before testing (<0.01 wt %).

Solid content. The solid content of the sample was determined according to GB/T 1725-1979:

(1) $$\mathrm{Solid}\%=\frac{\mathrm{Dried}\;\mathrm{sample}\;\mathrm{weight}}{\mathrm{Initial}\;\mathrm{sample}\;\mathrm{weight}}\times 100\%\;$$

Gel permeation chromatography (GPC). The Waters-515 gel permeation chromatograph (US WATERS Corporation) equipped with a Waters-2414 refractive index detector was used to measure the number-average molecular weight (*M*_n_) and weight-average molecular weight (*M*_w_) of the samples. THF was selected as eluent with polystyrene as calibration.

Viscosity. The viscosity of NWCPE dispersions was measured by DV-79 digital viscometer. The E-type rotor was selected and tested at 75 r min^−1^.

Water resistance. According to the national standard GB/T 1733-1993, the film was boiled in boiling deionized water for 2 h. The state of the film was observed.

Scratch experiment. The adhesion of the film coated on the tinplate was evaluated according to GB/T 9286-1998.

Pencil hardness. The pencil hardness of the film coated on the tinplate was tested according to GB/T 6739-2006.

Mechanical properties measurement. The tensile property of the film was tested according to the ASTM D 638 by using the Electronic Universal Testing Machine from MTS SYSTEMS (China) Co., Ltd. (Shanghai, China), at a crosshead speed of 10 mm min^−1^.

Light transmittance. The transmittance of the cross-linked NWCPE sample was tested by an ultraviolet−visible spectrometer (UV-3600, Shimadzu, Japan).

Scanning electron microscopy (SEM). The film was coated with gold at a voltage of 5 kV, and the sample was observed on a field emission scanning electron microscope (SEM, Zeiss Sigma, Germany).

Differential scanning calorimetry (DSC). TA Q2000 DSC Instrument (TA Instruments, New Castle, DE, USA) was used to test the glass-transition temperature (*T*_g_) and the measurement was carried out in a nitrogen atmosphere at a temperature range of −80 °C to 100 °C at a rate of 10 °C min^−1^.

Thermogravimetric analysis (TGA). The thermal stability of the sample was measured with a TG 209 F1 thermogravimetric analyzer from NETZSCH (Selb, Germany). The measurement was carried out in a nitrogen atmosphere at a temperature range of 30 °C to 650 °C and at the heating rate of 10 °C min^−1^.

## 3. Results and Discussion

In this work, a MA units containing linear polyester was synthesized by step-growth polycondensation. Following this, the hydrophilic polyether was grafted to the molecular skeleton as side chains through aza-Michael addition to prepare NWCPE.

### 3.1. Structure Characterizations

**Figure 1:**
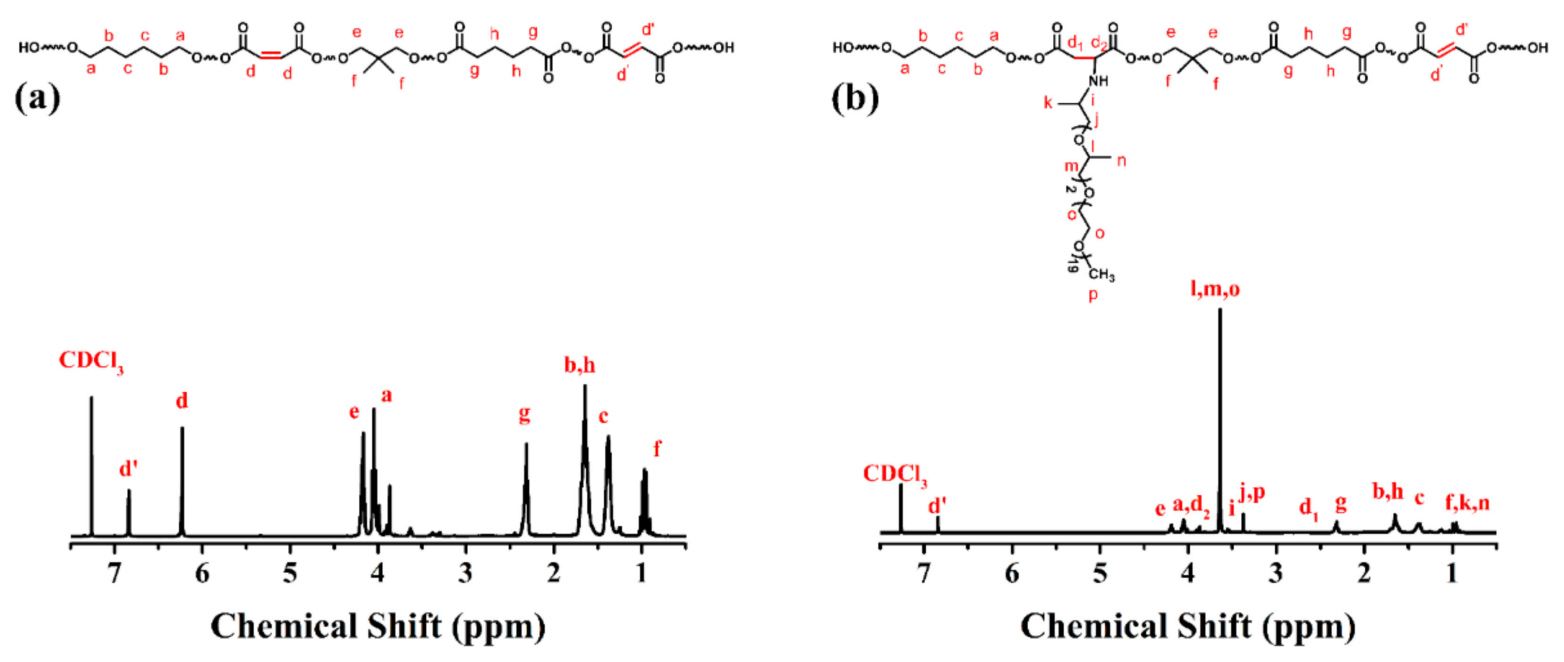
The ^1^H NMR of linear polyester (**a**), and comb-like polyester (**b**).

The ^1^H NMR data of Entry 3L and Entry 3C are given as a representative in Figure 1. The characteristic signal at 6.24 ppm originates from *cis* double bonds and the signal at 6.85 ppm belongs to *trans* double bonds. After the completion of the step-growth polycondensation, the integral area ratio of *cis* double bonds to *trans* double bonds was about 3:1, indicating that 25% of the *cis* units isomerized to *trans* units. The signal of the *cis* double bonds (δ = 6.24 ppm) disappeared and the signal of the *trans* double bonds (δ = 6.85 ppm) increased slightly after aza-Michael addition. The reason for this was that part of the *cis* units participated in the addition reaction and partially isomerized into the *trans* units [29]. The newly emerging signals at 4.02–3.98 ppm (−OOCCH_2_C***H***NHCH_2_−) and 2.69 ppm (−OCOC***H_2_***CHNHCH_2_−) belong to the reacted *cis* units’ proton. According to the integration analyses of the ratios between the two new signals and the trans signal peaks, the functionalization extent of the polyester was about 31%.

**Figure 2:**
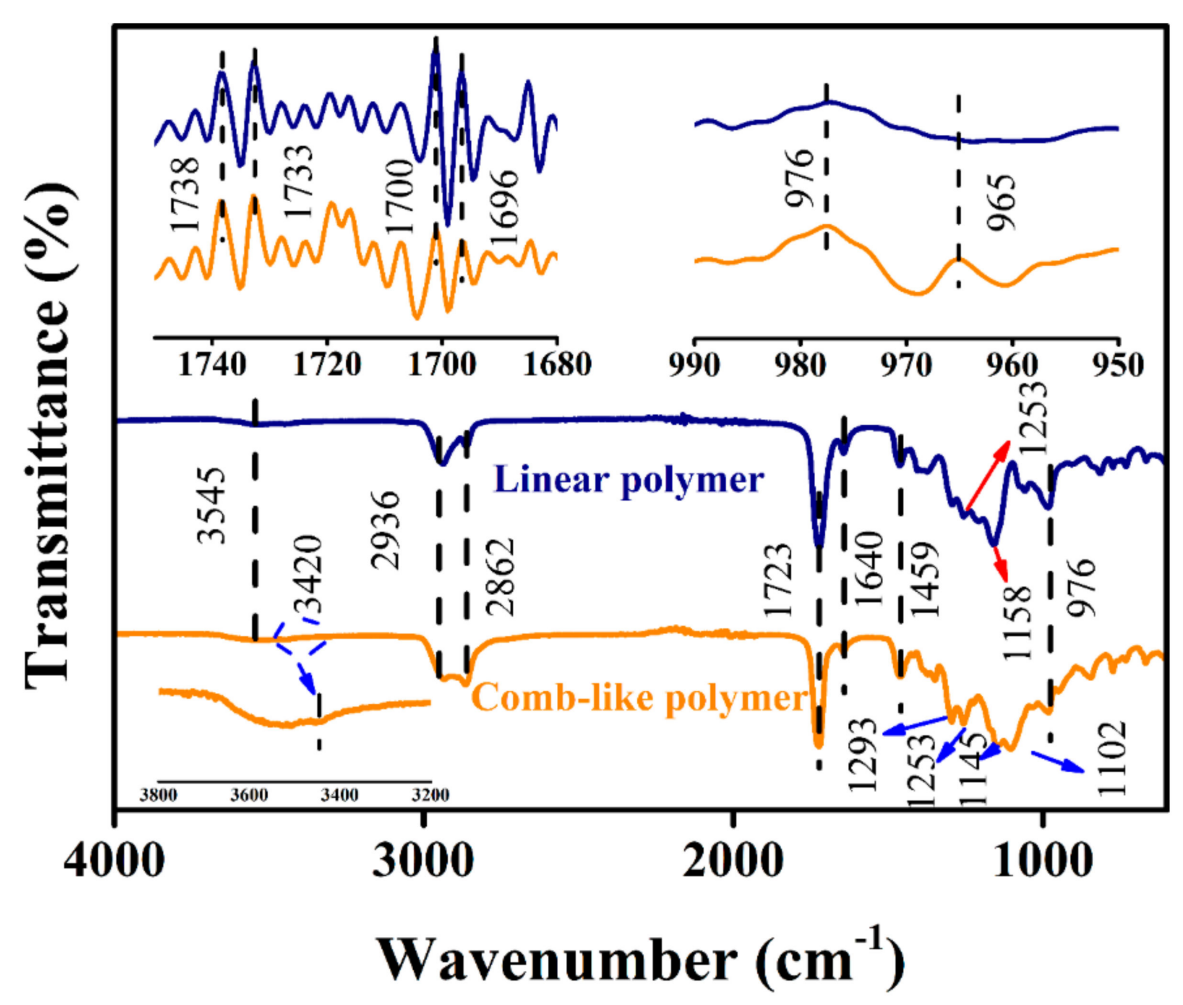
The FTIR spectrum and the second derivative spectrum of linear polyester and comb-like polyester.

The FTIR spectrum and the second derivative spectrum of both Entry 3L and Entry 3C are shown in Figure 2. The broad absorption peak at 3545 cm^−1^ is the stretching vibration peak of O−H, and the absorption peak at 3420 cm^−1^ in the spectrum of comb-like polyester is the stretching vibration peak of N−H. The stretching vibration peaks of −CH_3_ and −CH_2_ appear at 2936 cm^−1^ and 2862 cm^−1^, and the peak at 1459 cm^−1^ belongs to −CH_3_ and −CH_2_ bending vibration. The difference of the peak at 1723 cm^−1^ was amplified by the second derivative spectrum. The peak at 1740 cm^−1^ belongs to the carbonyl ester C=O, which is attached to C−C bonds. The peak that appears at 1700 cm^−1^ is ascribed to the carbonyl ester C=O attached to the C=C bonds. The two peaks at 1253 cm^−1^ and 1102 cm^−1^ belong to the asymmetric stretching vibration and symmetric stretching vibration of the carboxyl group C−O−C, respectively [30]. After the aza-Michael addition was accomplished, the ratio of the C−O−C peak intensity to the C=O peak absorption intensity increased, which indicated that the addition reaction was successful. The two peaks observed at 1640 cm^−1^ and 976 cm^−1^ are ascribed to the C=C tensile vibration and the *trans* C=C−H out-of-plane bending vibration, respectively. The peak intensity at 976 cm^−1^ was enhanced after aza-Michael addition, which indicates that cis units reduced in the addition process.

### 3.2. The Influence of Various Factors on Polyester and Polyester Dispersion

**Scheme 2:**
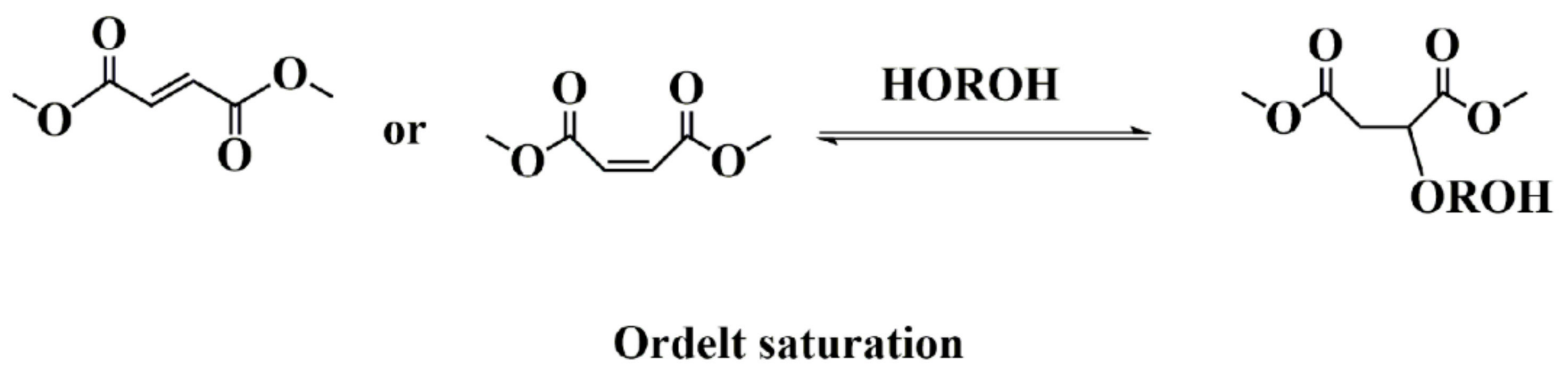
The Ordelt reaction, *cis* and *trans* C=C bonds saturate with diol.

The aza-Michael addition proceeds between a nucleophilic amine and an electron deficient alkene. In this work, the electron deficient alkene was the *cis* MA units, and the Jeffamine M-1000 acted as the nucleophilic amine. The *cis* double bonds of MA would isomerize into *trans* units in both polymerization and aza-Michael addition process, [31] but the amino-terminated hydrophilic polyether is more likely to react with *cis* double bonds in addition reaction [29,32]. The amount of the side chains was closely related to the *cis* MA units’ content on the molecular skeleton, which determined the hydrophilic nature and stability of the NWCPE dispersions. Moreover, in the polycondensation process a side reaction called Ordelt saturation took place between the hydroxyl group of the diol monomers and the double bonds of the unsaturated diacid. This interaction should be avoided, because it would lead to uncontrollable branching [33,34]. The routes of Ordelt saturation were described in Scheme 2. In the polycondensation process, the isomerization extent is related to various factors including reaction temperature and the type of catalyst. At the same time, the Ordelt saturation reaction is more likely to proceed at high temperatures. In addition, the properties of the NWCPE and the cross-linked film are related to the type and dosage of the monomers. Hence, it was worth studying the influence of the synthesis conditions such as catalyst type, reaction temperatures, and the dosage of different monomers on the polymer.

#### 3.2.1. Effect of the Catalyst Type

In previous works, various catalysts have been explored for the preparation of polyesters, including organometallic compounds Ti(OBu)_4_ [35,36], DBTDL [37], and acid TsOH [38,39]. In this paper, we investigated the influence of the catalyst type on the isomerization extent and molecular weight of the polyester. As shown in Table 2, when the Ti(OBu)_4_ was selected as the catalyst, higher molecular weight polyester was obtained, but the severe isomerization made it unsuitable for this system. Although the isomerization extent was well controlled with DBTDL as the catalyst, the molecular weight (2820 g mol^−1^) could be further improved. When TsOH was selected, the *M*_n_ of the synthesized polyester was about 5870 g mol^−1^, the isomerization extent was below 25%. Because the catalytic activity of Ti(OBu)_4_ was the higher than both TsOH and DBTDL, the Entry 2L was the largest in molecular weight, but the excessive degradation reactions of Ti(OBu)_4_ during polymerization process resulted the darkest color of the sample [37]. Moreover, the catalysts play an important in the isomerization process, which was usually used to manufacture fumarate from maleate [40], and the different isomerization extent using different catalysts may be related to the nucleophilicity [41] and rigidity [42] of the catalyst. In general, TsOH was more suitable as a polycondensation reaction catalyst in this work.

#### 3.2.2. Effect of the Reaction Temperature

**Figure 3:**
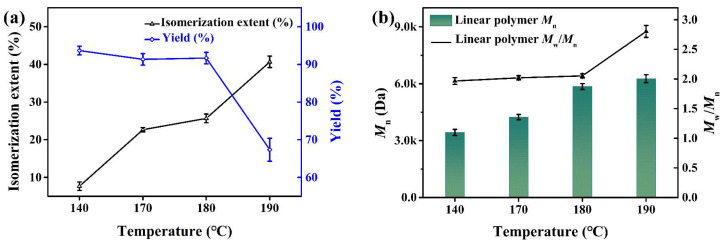
Effect of reaction temperatures on the isomerization extent and yield (**a**), *M*_n_ and *M*_w_/*M*_n_ (**b**) of polyester.

The reaction temperature is a significant yet complicated factor in polycondensation. High reaction temperatures were usually accompanied by some negative influence, including unwanted Ordelt saturation reaction and severe isomerization, whereas the polyester obtained at low temperature was usually lower in molecular weight [29,43]. As depicted in Figure 3, four samples were prepared under different reaction temperatures ranging from 140 °C to 190 °C. During the polymerization, the temperature must exceed 140 °C to maintain the molten state of the monomers. As shown in Figure 3a, the isomerization extent increased from 7% to 42% when the reaction temperature gradually increased from 140 °C to 190 °C. The driving force of the isomerization behavior is regarded to be that the *trans* isomer possesses higher energetic stability. The sufficiently thermodynamic driving force was obtained at a high temperature, which might lead to uncontrolled isomerization [44]. The *cis* units served as grafting sites for hydrophilic side chains, and its content was closely related to the water dispersibility of the synthesized polyester. An excessive degree of isomerization would result in insufficient grafting sites, leading to polyester that is difficult to disperse in water. In addition, even in the presence of antioxidants, when the temperature reached 190 °C, obvious gelation caused by Ordelt saturation reaction was observed, leading to the low yield. As shown in Figure 3b, the *M*_n_ of polyester ranged from 3410 g mol^−1^ to 6410 g mol^−1^ with the reaction temperature rising. The cause might be that the viscosity decreases with the increasing temperature, which accelerated the discharge of water. This drove the polymerization reaction forward further. In addition, 4-methoxyphenol was selected as a radical quencher to suppress the Ordelt saturation reaction in this manuscript. Satisfactorily, this was successful, as was confirmed by the relatively low molecular weight distribution value as shown in Figure 3b, which indicated the Ordelt saturation reaction was effectively suppressed [29]. On the whole, it was more reasonable to set the reaction temperature to 180 °C.

#### 3.2.3. The Influence of MA/HA Monomer Ratio

**Figure 4:**
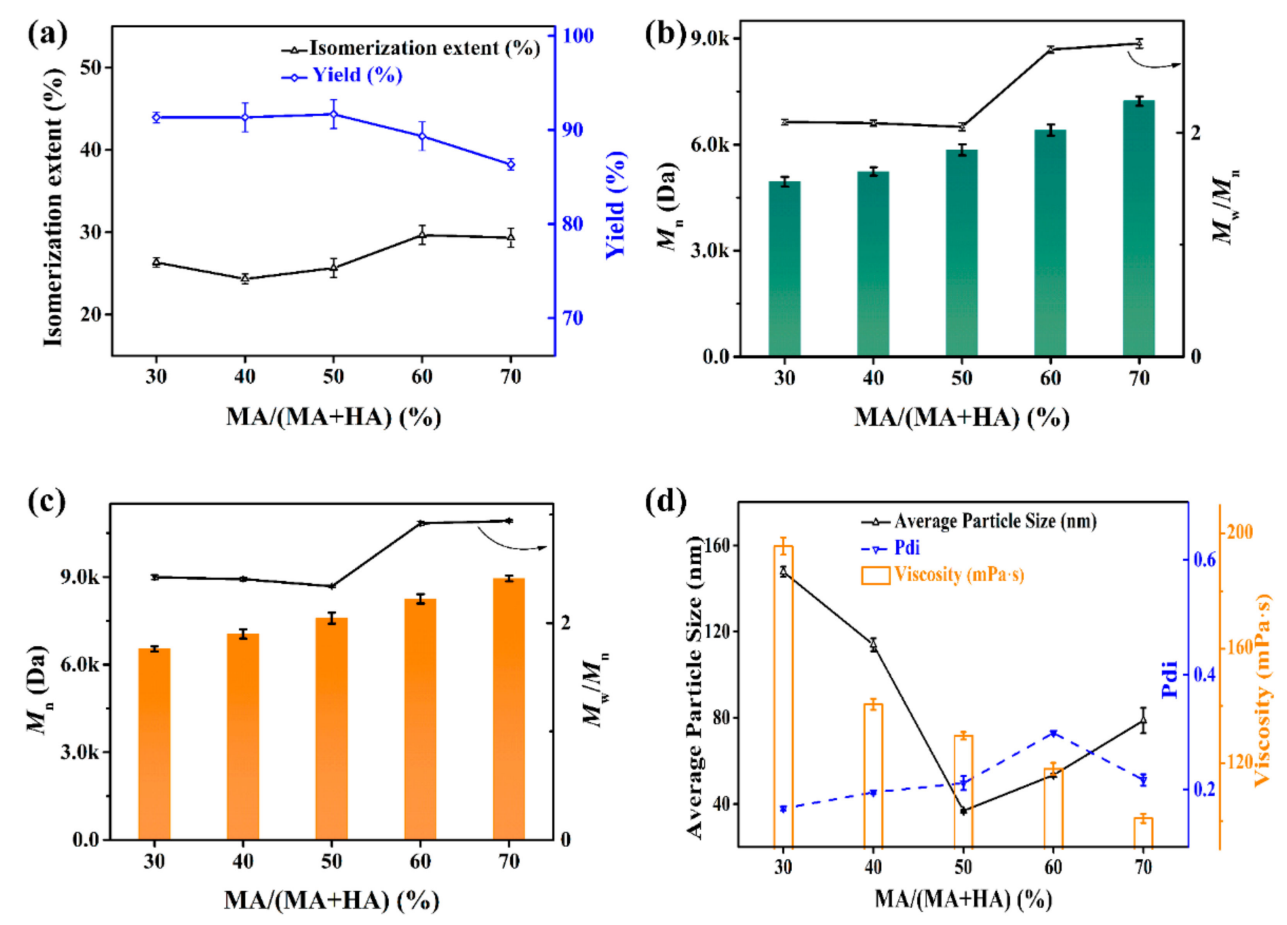
Isomerization extent and yield (**a**), *M*_n_ and *M*_w_/*M*_n_ of linear polyester (**b**) and comb-like polymer (**c**), particle size and viscosity (**d**) of polyester dispersions in different MA/(MA + HA).

The water dispersibility and storage stability of the NWCPE dispersions were affected by the density of side chains, which was related to the dosage of MA. The influence of the MA content was investigated with all other reaction parameters (the formulation of monomers, catalyst, inhibitor, and the reaction time), which were kept invariable. As we can see in Figure 4a, at the same reaction temperature the isomerization extent of the polyester was not significantly affected by the MA content, which stayed at 25−30%. As shown in Figure 4b, the *M*_n_ of the linear polyester increased from 4950 g mol^−1^ to 7230 g mol^−1^, with the content of MA increasing from 30% to 70%. This behavior might be related to the higher reactivity of MA as compared to HA during the polycondensation. In the same reaction time, it was found that the more reactive the monomer, the greater the polymerization extent, resulting in a larger molecular weight. All samples exhibited a reasonable polydispersity value, ranging between 2.05–2.79 for linear polyester. This indicates that no branched polymers were brought during the polymerization [33]. After functionalization was complete, the corresponding comb-like polyesters were obtained. As shown in Figure 4c, the *M*_n_ of the comb-like polyester changed from 6530 g mol^−1^ to 8950 g mol^−1^ with polydispersity ranging from 2.34 to 2.94. Because of the extra volume gained from the side chains, the *M*_n_ of comb-like polyesters increased slightly after the addition, and the polydispersity also became wider. In addition, the longer polyester possessed more side chains, which was related to the hydrophilic of NWCPE dispersion.

The self-dispersing ability in water of NWCPE dispersions with different MA content is shown in Figure 4d and Table 3. Due to the free hydrophilic side chains, Jeffamine M-1000 swelled well in water and provided good dispersion capability, and all of the polyesters were able to be self-dispersed in the water. With the content of MA improved from 30% to 50%, the amount of side chains increased from 3.52 to 4.94, the hydrophilicity of the polyester was significantly enhanced, and the particle size gradually decreased. However, with the further increase in the number of side chains, the particle size showed an increasing trend. The reason might be due to the following factors. As the number of side chains increased from 3.52 to 4.94, the hydrophilicity of the polymer increased. At the same time, owing to the increase in side chains, the intermolecular repulsion increased and the intermolecular aggregation decreased. All of these led to a decrease in particle size. On the other hand, as the MA dosage continued to increase, the molecular weight of the polymer increased slightly. Meanwhile, the polyester particles were greatly softened with an increase in flexible polyether-amine segments. The soft particles tended to coalesce under the external shear force during the emulsification process [45,46]. Hence, the particle size of dispersions decreased and then increased with the increase in the number of side chains. The storage stability of dispersions is related to the average particle size and viscosity [47]. The prepared dispersions were metastable systems because of the small average particle size [48]. In addition, with the increase in the density of side chains, the viscosity of the dispersion decreased. The reason might be that, with the increase in the number of side chains, the hydrophilic components of the polyester increased, and the hydrodynamic volume of particles decreased [49,50,51]. When the number of side chains exceeded 4.94, the viscosity was lower than 120 mPa·s and the particle size of the dispersion was below 100 nm. According to Stokes’ law, dispersions with small particle size and low viscosity are more prone to precipitate [47]. As a result, the storage stability of the dispersions deteriorated seriously and obvious flocculation was observed in less than 30 days. As for the coating properties, with the MA content increased from 30% to 70%, the pencil hardness of cured films was maintained at B, the adhesive property was kept at level 1, and all cured films exhibited good water resistance. In summation, when the content of MA was 50%, the prepared NWCPE dispersion and cross-linked film possessed good performance.

#### 3.2.4. The Influence of the Rigid Monomer Content

**Figure 5:**
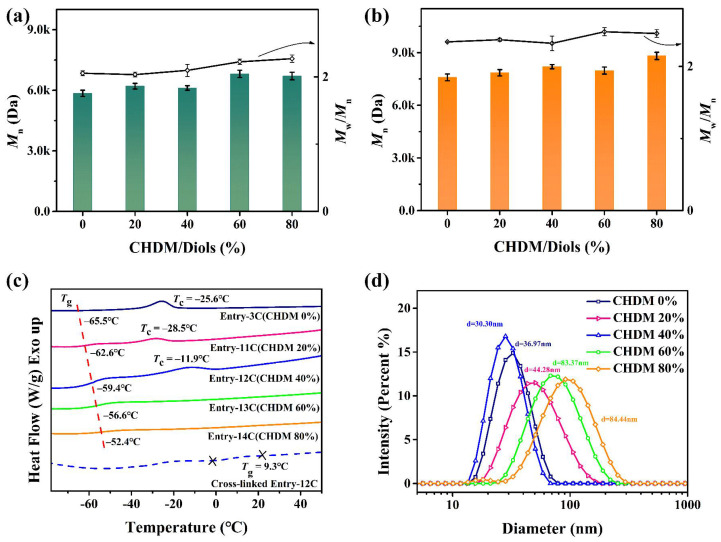
Molecular weight and polydispersity for linear polyester (**a**) and comb-like polyester (**b**), DSC curves of comb-like polyester and part of cross-linked polyester (**c**), and average particle size of dispersions (**d**) in different CHDM content.

Since our objective is to synthesize a co-polyester with good comprehensive properties, mechanical properties are of great concern to us. As such, the toughness monomer CHDM was introduced into the molecular skeleton [52,53,54]. Although the transformation of isomerization is mainly affected by the steric hindrance effect of the polymer, the synthesized polyesters exhibited almost no difference in isomerization extent (all were around 25%) with the increase in CHDM dosage. The reason for this might be that the two primary hydroxyl groups of CHDM are widely separated [31]. The result indicated that this method is universal and applicable to a wide variety of diols. As can be gathered from Figure 5a,b, the *M*_n_ of all linear polymer ranged from 5850 g mol^−1^ to 6810 g mol^−1^ with the polydispersity ranging between 2.03–2.27, the *M*_n_ of comb-like polymer ranged from 7590 g mol^−1^ to 8810 g mol^−1^, with the polydispersity ranging from 2.32 to 2.48. All synthesized polyesters were similar in molecular weight and polydispersity, indicating that the step-growth polycondensation of the system was not significantly affected when the HG was replaced with CHDM.

As shown in Figure 5c, all synthesized comb-like polyesters only showed one glass transition temperature, indicating that the polyesters were random co-polyesters. As the content of rigid monomer CHDM increased, the *T*_g_ of the polyester rose slightly. Furthermore, the crystal behavior existed in some samples, and it was found that the higher the CHDM content, the higher the crystallization temperature. The mobility of the NWCPE molecule was limited when the soft monomer was replaced by more than 60%. Therefore, the crystal behavior was not observed in samples CHDM 60% and CHDM 80% [55]. After the NWCPE was cross-linked with the WHDIT, the *T*_g_ increased significantly, and, due to further restricted in the movement of polymer chains, no crystallization melting behavior was observed.

As we can see from Figure 5d, after dispersing into water the average particle size of NWCPE dispersions ranged from 30.3 nm to 84.4 nm with the pdi value below 0.25. When the CHDM was introduced to the polyester sparingly, there was nearly no difference in average particle size, but the polyester particle size increased significantly when the CHDM content exceeded 40%. It might be that when the soft monomer HA was largely replaced by the more rigid monomer CHDM, the movement of the polymer chains was limited, resulting in poorer swelling of the NWCPE. Owning to the similar molecular weight and the density of side chains, the hydrodynamic volume of the NWCPEs in water with different CHDM content was relatively close, and the viscosity of all the dispersions was at about 130 mPa·s [56]. The storage stability would be similar naturally when the dispersions were closely in the particle size and viscosity.

**Figure 6:**
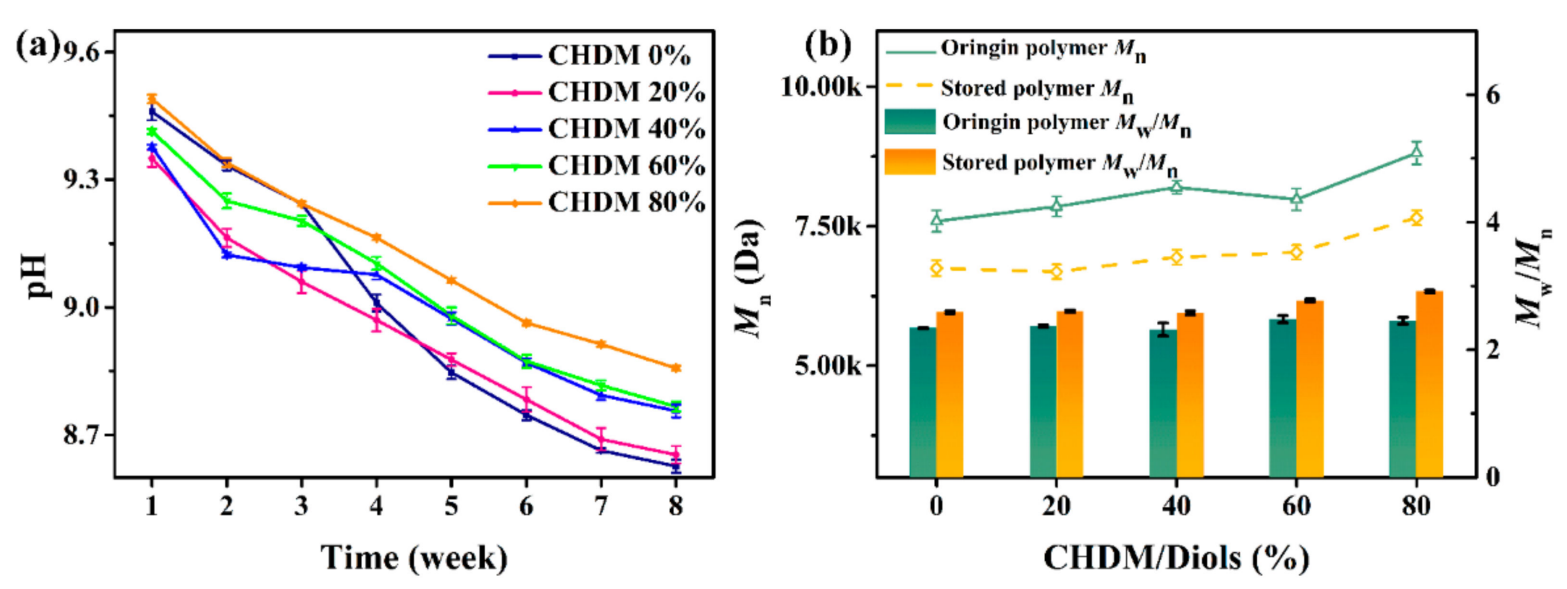
The ΔpH of dispersions (**a**), and Δ*M*_n_ of comb-like polyester after storage for 6 months (**b**) in different CHDM content.

As shown in Figure 6a, all polyester dispersions were alkaline because of the polyether-amine side chains, and the pH value of dispersions decreased during storage. In this work, the ΔpH of prepared dispersions was below 0.8 after 2 months of storage at room temperature. As mentioned in previous work, the pH of polyester dispersions changed above 1.5 after just 20 days of storage [57]. As we can see from Figure 6b, after 6 months of storage, the *M*_n_ of the polymer just decreased by a few hundred Da, and the change was less than 10%, while the *M*_n_ of polyester which, in other work, dropped by 40% after only being stored for one week [58]. This result indicates that the prepared NWCPE dispersions possess stronger hydrolysis resistance than waterborne ionic polyester. Furthermore, after the CHDM was utilized as a monomer, the decline in pH value and *M*_n_ were arrested, which was also related to the hydrolysis resistance of CHDM.

The hardness of the coatings is related to the cross-linking density of networks and the chemical structure of the prepolymers [59]. As displayed in Table 4, the pencil hardness of the cross-linked coating increased with the increase in the CHDM content. The reason might be that the rigidity and the intermolecular force of the cured polyester increased with the increasing rigidity of diol. However, when the CHDM content reached 40%, the improvement of the rigid monomer dosage showed little effect on the hardness of the film. After the cross-linking process was accomplished, many urethane bonds were formed on the molecule, which grants that all cross-linked coatings exhibited good adhesion on the tinplate substrate. With the increasing dosage of CHDM, the molecular rigidity increased as well. The aliphatic chain length decreased compared to HA, but due to the steric hindrance of the side chains, the internal stress displayed almost no change [60]. As a result, the adhesion did not decrease with the utilization of CHDM. Because of the hydrophilic polar groups, the waterborne coatings are more sensitive to moisture, hence demonstrating that water resistance is an important factor in determining their application. Because the hydrophilic segments of the waterborne non-ionic polymer acted as side chains, all cross-linked films displayed good water resistance.

### 3.3. Thermogravimetric Analysis

**Figure 7:**
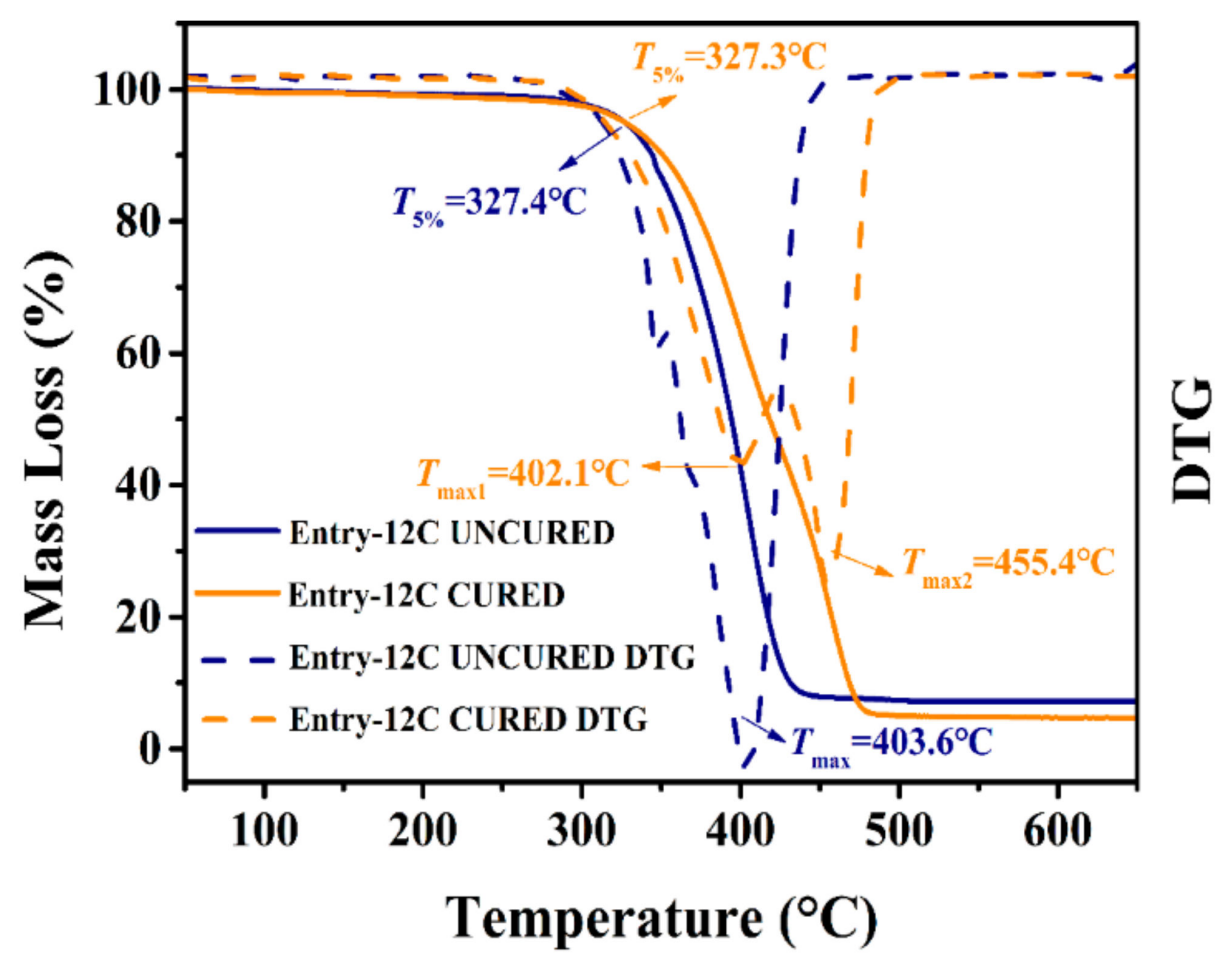
TG and DTG of NWCPE and cured polyester film.

The thermal degradation behaviors and DTG curves of Entry 12C are shown in Figure 7, and some crucial data are also displayed. The degradation curve of the cross-linked polyester in the first step was almost the same as the pure polyester, which was reflected by the same 5% weight loss temperature (327 °C) and the maximum weight loss rate temperature (403 °C). This step degradation was attributed to the ester and aliphatic moieties. The Tmax2 (455 °C) was observed in the cross-linked sample, which indicated the cross-linked polyester degraded by a multistep process. The reason might be that the hard segment urethane bonds formed after cross-linking, which reduced the migration of the chain segments. The thermal stability of the material was enhanced after cross-linking [61].

### 3.4. Tensile Properties of the Cross-Linked NWCPE Films

**Figure 8:**
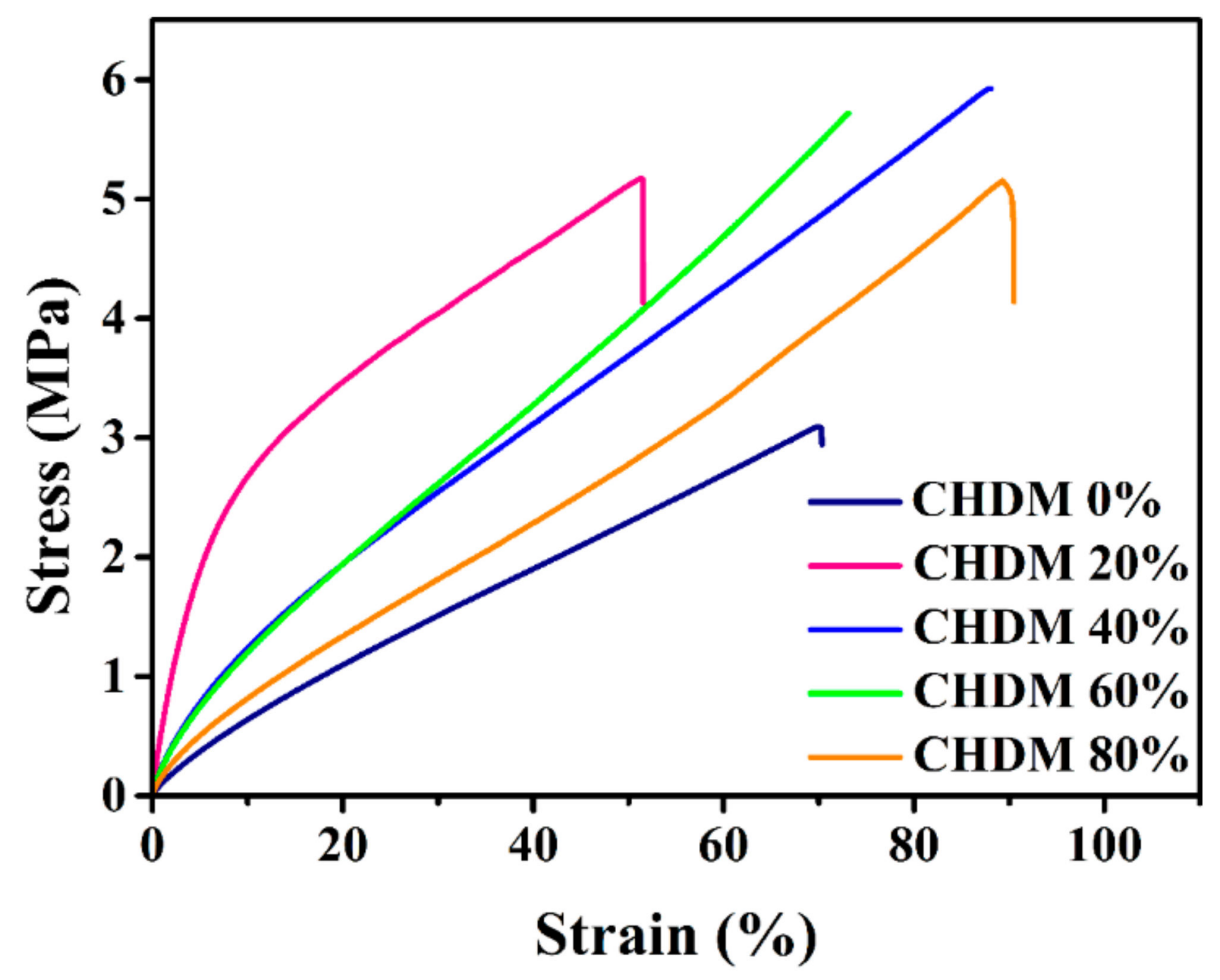
Stress-strain profiles of cross-linked films.

The tensile properties of cross-linked NWCPE films were summarized in Table 5, and the stress–strain cures were listed in Figure 8. Mechanical properties are crucial for polymer materials which directly affect their industrial applications. The tensile strength increased from 3.1 MPa to 5.9 MPa after CHDM being introduced into the molecule. But when the content of CHDM reached 40%, the strength of the polymer no longer increased with the increase in the CHDM content. In addition, the toughness even decreased slightly due to the excessively high content of this rigid monomer. The result may be related to the following factors. First, the utilization of CHDM enhanced the rigidity of the polymer which could restrict the movement of the molecular chains, and the intermolecular force was increased, resulting in improved mechanical properties. Secondly, the molecular weight of the polymers were slightly different. Polyester with minor *M*_n_ contained more terminal hydroxyl groups in the same mass, resulting in a slightly larger cross-linking density during the curing process. The stress–strain curves of all of the films showed typical plastic deformation, and the elongation at the break (ε) of the films were all basically above 70%. The cause might be the following two factors. First, the synthesized polyester has low crystallinity, leading to high mobility between molecular chains. Secondly, the polyether-amine side chains are similar to a spring and increased the ductility of the molecular. The tensile properties of cured NWCPE were better than some cured ionic waterborne polyesters, some of which were about 2–3 MPa [3,62,63].

### 3.5. The Morphology and Light Transmittance of the Cured Film

**Figure 9:**
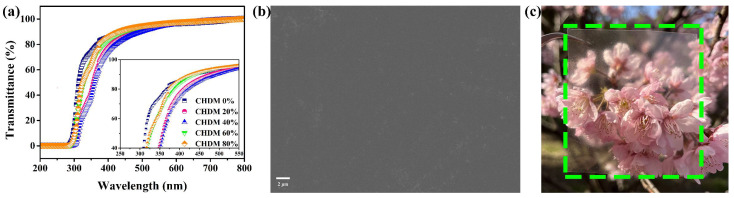
The light transmittance (**a**), SEM image (**b**), and photograph (**c**) of cross-linked films.

As shown in Figure 9a,c, the neat cross-linked film was transparent, and, at the entire visible wavelength range, the light transmittance of the film was high. In addition, owing to the absence of emulsifier, no surfactant molecules migration during the film-forming process was observed, and the surface of the film was smooth and flat according to Figure 9b [64]. At the same cross-linking density, the CHDM content exerted almost no influence on the transmittance of the film. The SEM and photographic images of cured Entry 12C (CHDM 40%) were taken as examples. The high transparency was attributed to the following reasons. First, the synthesized NWCPEs have good compatibility with the curing agent WHDIT and there was not significant macrophase separation during the cross-linking procedure. Second, the surface of the film was smooth, which was hard for scattering light [65]. As such, the low crystallinity of the WCPE may also contribute to the high transmittance of the film.

## 4. Conclusions

In this paper, MA, HA, CHDM, HG, and NPG were first used as monomers to synthesize linear polyesters by step-growth polycondensation. After this, the hydrophilic segment Jeffamine M-1000 was grafted to the polymer backbone by aza-Michael addition to prepare the NWCPE. The optimal reaction conditions were verified as TsOH as the selected catalyst, a reaction temperature of 180 °C, a MA/HA ratio of 5:5, and the content of the rigid monomer CHDM at 40%. In this condition, NWCPE with a molecular weight of about 8000 g mol^−1^ was prepared. After dispersing into water, the dispersion with a solid content of 40% and the particle size was appropriate with narrow distribution. The dispersion was good in storage stability and remained stable after 6 months. The good hydrolysis resistance was identified by the slight drop in pH after two months and little change in *M*_n_ of the dispersion. After cross-linking with the curing agent, a transparent film with good mechanical properties and water resistance was obtained. The hardness of the film was H, and the adhesion level on the tinplate reaches level 0. The tensile strength of the film was 5.9 MPa, and the *ε* was about 88%. This work provides ideas for the synthesis and functionalization of waterborne polyesters. These synthesized polymers have wide application prospects in environmentally friendly polymer materials.

## Figures and Tables

**Table 1:** The formulation and synthesis condition of NWCPEs.

Sample ^(a)^	Catalyst	Reaction Temperature (°C)	Diacid Dosage (mol)		Diol Dosage (mol)			Functionality ^(b)^
			MA	HA	CHDM	HG	NPG	
Entry-1L	DBTDL	180	0.05	0.05	0	0.084	0.021	/
Entry-2L	Ti(OBu)_4_	180	0.05	0.05	0	0.084	0.021	/
Entry-3L	TsOH	180	0.05	0.05	0	0.084	0.021	/
Entry-3C								4.94
Entry-4L	TsOH	140	0.05	0.05	0	0.084	0.021	/
Entry-5L	TsOH	170	0.05	0.05	0	0.084	0.021	/
Entry-6L	TsOH	190	0.05	0.05	0	0.084	0.021	/
Entry-7L	TsOH	180	0.03	0.07	0	0.084	0.021	/
Entry-7C								3.52
Entry-8L	TsOH	180	0.04	0.06	0	0.084	0.021	/
Entry-8C								4.16
Entry-9L	TsOH	180	0.06	0.04	0	0.084	0.021	/
Entry-9C								5.63
Entry-10L	TsOH	180	0.07	0.03	0	0.084	0.021	/
Entry-10C								7.48
Entry-11L	TsOH	180	0.05	0.05	0.021	0.063	0.021	/
Entry-11C								4.48
Entry-12L	TsOH	180	0.05	0.05	0.042	0.042	0.021	/
Entry-12C								4.82
Entry-13L	TsOH	180	0.05	0.05	0.063	0.021	0.021	/
Entry-13C								5.15
Entry-14L	TsOH	180	0.05	0.05	0.084	0	0.021	/
Entry-14C								5.21

(a) The linear and comb-like polyesters in same formula were distinguished by suffix letters: Entry-L represented the linear polyester and Entry-C represented the comb-like polyester; (b) the functionality (i.e., the number of side chains on a molecular chain) was calculated with 1H NMR spectroscopy and GPC data. (The calculation method was based on the protons of HA units (-OOCC***H_2_***(CH_2_)_2_C***H_2_***COO-) and the protons of the residual *trans* MA units.)

**Table 2:** Effect of catalyst type on the polymerization and properties of polyester.

Sample	Catalyst	*cis:trans*	*M*_n_ (g mol^−1^)	*M*_w_/*M*_n_	Appearance
Entry-1L	DBTDL	83:17	2820	2.12	Light yellow transparent
Entry-2L	Ti(OBu)_4_	62:38	14330	2.25	Dark brown transparent
Entry-3L	TsOH	75:25	5870	2.05	Yellow transparent

**Table 3:** The properties of NWCPE dispersions and cured films with different MA dosage.

Sample	MA Content	Dispersion Appearance	Pencil Hardness	Adhesion	Water Resistance	Storage Stability
Entry-7C	30%	Milky white, untransparent	B	1°	Transparent	>180 d
Entry-8C	40%	Light yellow, transparent	B	1°	Transparent	>180 d
Entry-3C	50%	Light yellow, transparent	B	1°	Transparent	>180 d
Entry-9C	60%	Flocculation	/	/	/	<30 d
Entry-10C	70%	Flocculation	/	/	/	<30 d

**Table 4:** The properties of NWCPE dispersions and cured films with different CHDM dosage.

Sample	CHDM Content	Dispersion Appearance	Pencil Hardness	Adhesion	Water Resistance	Storage Stability
Entry-3C	0%	Light yellow, transparent	B	1°	Transparent	>180 d
Entry-11C	20%	Light yellow, transparent	HB	1°	Transparent	>180 d
Entry-12C	40%	Light yellow, transparent	H	0°	Transparent	>180 d
Entry-13C	60%	Light yellow, transparent	H	0°	Transparent	>180 d
Entry-14C	80%	Dark yellow, transparent	H	0°	Transparent	>180 d

**Table 5:** Tensile properties of cross-linked films.

CHDM Content	0%	20%	40%	60%	80%
*σ* (MPa)	3.1 ± 0.2	5.2 ± 0.2	5.9 ± 0.2	5.8 ± 0.3	5.2 ± 0.3
*Ε* (MPa)	4.0 ± 0.4	10.1 ± 0.3	5.8 ± 0.5	6.7 ± 0.8	4.8 ± 0.7
*ε* (%)	70.3 ± 0.8	51.6 ± 0.6	88.1 ± 0.4	73.2 ± 0.9	90.5 ± 1.2

## Data Availability

Not applicable.
